# A single fast Hebbian-like process enabling one-shot class addition in deep neural networks without backbone modification

**DOI:** 10.3389/fnins.2024.1344114

**Published:** 2024-06-12

**Authors:** Kazufumi Hosoda, Keigo Nishida, Shigeto Seno, Tomohiro Mashita, Hideki Kashioka, Izumi Ohzawa

**Affiliations:** ^1^Center for Information and Neural Networks, Advanced ICT Research Institute, National Institute of Information and Communications Technology, Suita, Japan; ^2^Life and Medical Sciences Area, Health Sciences Discipline, Kobe University, Kobe, Japan; ^3^Laboratory for Computational Molecular Design, RIKEN Center for Biosystems Dynamics Research, Suita, Japan; ^4^Department of Bioinformatic Engineering, Graduate School of Information Science and Technology, Osaka University, Suita, Japan; ^5^Cybermedia Center, Osaka University, Suita, Osaka, Japan

**Keywords:** one-shot learning, one-shot class addition, Hebbian theory, fast Hebbian plasticity, weight imprinting, quantile normalization, non-parametric method

## Abstract

One-shot learning, the ability to learn a new concept from a single instance, is a distinctive brain function that has garnered substantial interest in machine learning. While modeling physiological mechanisms poses challenges, advancements in artificial neural networks have led to performances in specific tasks that rival human capabilities. Proposing one-shot learning methods with these advancements, especially those involving simple mechanisms, not only enhance technological development but also contribute to neuroscience by proposing functionally valid hypotheses. Among the simplest methods for one-shot class addition with deep learning image classifiers is “weight imprinting,” which uses neural activity from a new class image data as the corresponding new synaptic weights. Despite its simplicity, its relevance to neuroscience is ambiguous, and it often interferes with original image classification, which is a significant drawback in practical applications. This study introduces a novel interpretation where a part of the weight imprinting process aligns with the Hebbian rule. We show that a single Hebbian-like process enables pre-trained deep learning image classifiers to perform one-shot class addition without any modification to the original classifier's backbone. Using non-parametric normalization to mimic brain's fast Hebbian plasticity significantly reduces the interference observed in previous methods. Our method is one of the simplest and most practical for one-shot class addition tasks, and its reliance on a single fast Hebbian-like process contributes valuable insights to neuroscience hypotheses.

## 1 Introduction

As well-known, artificial neural networks (ANNs) were initially inspired by biological neural networks in the animal brain (McCulloch and Pitts, 1943). Subsequently, Deep Neural Networks (DNNs) have achieved significant success in computer vision (Simonyan and Zisserman, 2015; He et al., 2016). However, several tasks, which are relatively easy for humans, remain challenging for current DNNs (Lake et al., 2017). One-shot learning, for instance, is a notable example of such tasks (Fei-Fei et al., 2006; Lake et al., 2015; Brea and Gerstner, 2016; Cowley et al., 2022).

Humans can integrate a new concept into their knowledge from just a single input image, experiencing little interference with prior memories. In contrast, DNNs struggle with this task unless they undergo specific additional optimization. Proposing a simple one-shot learning mechanism in DNNs could enhance understanding of the brain and improve practical applications. Consider, for example, an ImageNet model (Deng et al., 2009; Russakovsky et al., 2015) that has been pre-trained on 1,000 classes and then learns a new class “baby” from a single image, without any additional training optimization or modifications to the original DNN backbone. If such one-shot class addition is achievable through a simple brain-based process, it would be beneficial for both understanding the brain and advancing machine learning applications, which is the focus of this study.

Extensive research has been conducted on the brain mechanisms underlying one-shot or fast learning (Piette et al., 2020). In the brain, new information is typically more effectively retained when linked to existing knowledge (Tse et al., 2011; Kesteren et al., 2012), a principle that also applies to one-shot learning (Achterberg et al., 2022). It is known that the hippocampus switches between fast learning and general slow incremental learning (Lee et al., 2015; Weaver, 2015), and that both the frontal lobe and hippocampus contribute to fast learning (Preston and Eichenbaum, 2013; Emberly and Seamans, 2020). It has also been suggested that fast learning does not necessarily require the hippocampus and is possible through mechanisms similar to general slow learning (Hebscher et al., 2019). Although the classical Hebbian framework assumes repetition of a specific activity pattern (Hebb, 1949), Hebbian long-term potentiation is known to rapidly occur with a small number of spikes (Froemke et al., 2006). Such “fast Hebbian plasticity” is no longer an unrealistic hypothesis but recognized as a hot topic in neuroscience (Lansner et al., 2023). Therefore, while the entire brain's mechanisms are complex and yet unclear, it appears likely that at least a fast Hebbian process plays a significant role.

From a machine learning perspective, typical approaches for one- or few-shot image classification include metric learning, data augmentation, and meta-learning. Metric learning reduces distances between similar class data in feature space and increases it for different classes (Weinberger and Saul, 2009; Snell et al., 2017; Kaya and Bilge, 2019), akin to the brain's process of minimizing interference in new learning, thought to be managed by the hippocampus (McCloskey and Cohen, 1989; McClelland et al., 1995). Data augmentation techniques expand training data through generative methods (Goodfellow et al., 2014; Vinyals et al., 2016; Schwartz et al., 2018), potentially enabling fast learning by leveraging previous expectations or binding patterns (Smolensky, 1990; Friston, 2010). Meta-learning, which trains systems in learning methodologies (Andrychowicz et al., 2016; Finn et al., 2017; Huisman et al., 2021), has been employed to model the prefrontal cortex (Wang et al., 2018) and can be enhanced by incorporating Hebbian learning (Munkhdalai and Trischler, 2018).

However, most methods prioritize high performance and involve specific optimizations, diverging from simpler models that might better represent natural processes. Additionally, these optimizations often require extensive user skills and incur higher computational costs for tuning parameters and hyperparameters. Furthermore, many methods focus on learning from scratch or transfer learning, deviating from how the brain is thought to perform one-shot learning by utilizing vast existing knowledge.

Considering machine learning tasks from the perspective of the brain's one-shot learning, the emphasis should be on adding new classes to well-trained DNNs rather than learning from scratch or transfer learning. Studies have shown that well-trained DNNs are capable of identifying data deviating from the training distribution, which is known as out-of-distribution detection (Lakshminarayanan et al., 2017; Fort et al., 2021), suggesting that an effective representation for unknown images exists in the hidden multi-dimensional space.

Indeed, the *weight imprinting* method, proposed by Qi et al. (2018), enables the addition of novel classes to Convolutional Neural Networks (CNNs) using the final dense layer input of a new-class image without additional training. This method, requiring minimal CNN architecture modifications, can achieve reasonable accuracy in one-shot image classification tasks (for example, achieving 21% accuracy for novel-class images when adding 100 new classes to the original 100 in the CUB-200-2011 dataset). However, the connection between the weight imprinting method and the brain's mechanisms remains unclear and unexplored. Furthermore, from an application standpoint, Qi's weight imprinting method can interfere with original image classifications, potentially causing significant drawbacks in practical use (see Section 4, which indicates that Qi's method poses issues in CNNs but not in vision transformers). Note that Khan et al. (2021) demonstrated the use of weight imprinting for vision transformers, without addressing its issues on CNNs.

**Figure 1:**
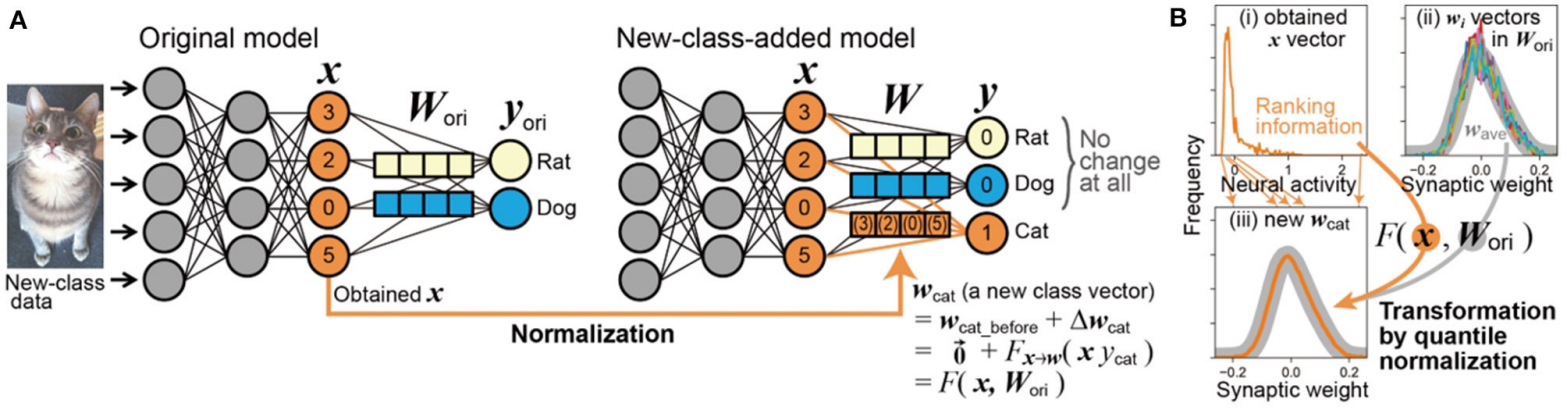
Scheme of DONE. **(A)** The neural activity input of the final dense layer (orange ***x*** vector in the original model) obtained from new-class data (an image of a cat) is directly used for the transformation into the new-class vector (orange ***w***_cat_) in the new weight matrix (***W***) without any modification to the backbone model. **(B)** Example of transformation from ***x*** to ***w***_cat_, with actual distribution data when the backbone DNN is EfficientNet-B0. See text for detailed explanation.

In this study, we investigated the task of one-shot class addition in vision, where new classes were added to a pre-trained DNN for image classification. Our approach included (i) proposing a novel interpretation in which a part of Qi's weight imprinting method can be understood as a Hebbian-like process; (ii) demonstrating that one-shot learning is achievable using only this Hebbian-like process alone with an accuracy ~80% of the original classification. We have termed this streamlined weight imprinting method without backbone modification as “Direct ONE-shot learning” (DONE). Specifically, as shown in Figure 1A, DONE directly transforms the input of the final dense layer (***x*** vector in the figure), which is obtained from a single image belonging to a new class (e.g., a cat), into the corresponding weight vector for this new class (***w***_cat_, a row vector in the weight matrix ***W***). This process adds weight vectors for new classes without altering the backbone DNN or the original weight matrix ***W***_ori_. Additionally, we (iii) found that aligning the single Hebbian-like process more closely with brain mechanisms, specifically through quantile normalization, mitigates the severe interference observed in Qi's method. We then (iv) pinpointed the cause of interference in Qi's method, uncovering a notable distinction between vision transformers and CNNs. Our findings that typical DNNs can facilitate one-shot learning through a simple brain-based process contribute to both neuroscience and practical applications. While it is still uncertain whether this process occurs in the brain, it offers valuable hypotheses for future neuroscience research. At least, this study suggests that one-shot learning is no longer beyond the reach of DNNs.

## 2 Weight imprintings and Hebbian interpretation

### 2.1 Qi's weight imprinting

Weight imprinting, a class-addition method that emerged from the context of metric learning (Qi et al., 2018), does not require any optimization algorithms, making it accessible for users without specialized machine learning knowledge. Here, we detail the specific procedure employed in the previous Qi's method. Consider the final dense layer of a general classification DNN model: typically, the output vector ***y*** = (*y*_1_, ⋯ , *y*_*N*_) of the final dense layer indicates the likelihood that an image belongs to each of the *N* classes. It is computed from the input vector ***x*** = (*x*_1_, ⋯ , *x*_*M*_), the weight matrix ***W*** (*N*×*M*), and the bias vector ***b*** = (*b*_1_, ⋯ , *b*_*N*_). For the *i*-th class (*i* = 1, 2, ⋯ , *N*), a scalar *y*_*i*_ is calculated using the corresponding weight vector ***w***_*i*_ = (*w*_*i*1_, ⋯ , *w*_*iM*_) (the *i*-th row of the ***W*** matrix) and the bias scalar *b*_*i*_ as follows:


(1) $$\begin{array}{l} y_{i}=x\cdot w_{i}+b_{i}=||x|{|}_{2}||w_{i}|{|}_{2}\cos\theta +b_{i}, \end{array}$$

where the cosine similarity, representing the similarity between vectors ***x*** and ***w***_*i*_ irrespective of their magnitudes, is a component of the model's objective function.

The cosine similarity (in Equation 1) reaches its maximum value of one when ***x*** and ***w***_*i*_ are perfectly aligned. Therefore, if an ***x*** vector is directly used as the weight vector for a new *j*-th class ***w***_*j*_ (*j* = *N*+1, ⋯ ), the cosine similarity for the *j*-th class will be high when another ***x*** vector with a similar orientation is presented.

Qi's method employs cosine similarity as the sole metric for its objective function. The procedure involves the following three key modifications to the backbone DNN models:

- **Modification 1:** Addition of an *L*_2_ normalization layer before the final dense layer to transform ***x*** into a unit vector, i.e., ||***x***||_2_ = 1
- **Modification 2:** Normalization of all ***w***_*i*_ vectors to become unit vectors, ensuring ||***w***_*i*_||_2_ = 1 for all *i*.
- **Modification 3:** Elimination of all bias values *b*_*i*_, i.e., ***b*** = 0.

Then, the weight imprinting process is defined as follows:

- **Weightimprinting:** The *L*_2_-normalized final-dense-layer input from a new-class image ***x***_new_ is used as the weight vector for the new class ***w***_*j*_, i.e., ***w***_*j*_ = ***x***_new_.

Qi's method is simpler than other one-shot learning methods that require additional optimization. However, it still involves some modifications to the backbone DNN, including changes in the objective function. The appropriateness of such modifications depends on the specific context. Generally, avoiding modifications is advisable when not essential, to prevent unnecessary complications and potential interference with the original classification, given that the backbone DNN is typically already well-optimized for a certain function. Most importantly, in the context of modeling the brain, a simpler approach is often more desirable. Therefore, avoiding modifications is preferable in pursuit of a more brain-like model.

Furthermore, Qi's method does not address the statistical discrepancies between ***x*** and ***w***_*j*_, which limits the range of applicable backbone DNNs (for more details, see Section 4). While various studies have developed Qi's method further, making it more complex and adaptable (Dhillon et al., 2019; Li et al., 2021; Passalis et al., 2021; Cristovao et al., 2022; Zhu et al., 2022), to the best of our knowledge, none have sought to simplify it or effectively tackle the transformation problem from ***x*** to ***w***_*j*_.

### 2.2 Interpretation of weight imprinting via Hebbian theory

A specific process within weight imprinting can be interpreted as a change in synaptic weight according to Hebbian theory. As shown in Figure 1A, in weight imprinting, we can consider that the new weight vector ***w***_cat_ initially emerges from a state of zero, and thus, it is equivalent to its change, i.e., $w_{\text{cat}}=\vec{0}+\Delta w_{\text{cat}}=\Delta w_{\text{cat}}$.

Hebbian theory pertains to the changes in synaptic weight, Δ***w***_cat_, and posits that a synaptic weight is strengthened when both presynaptic and postsynaptic neurons are active simultaneously. When a single image of a new class (cat) is presented as visual input, certain presynaptic neurons ***x*** become active. Simultaneously, the postsynaptic neuron corresponding to the cat becomes active (e.g., *y*_cat_ = 1), while the postsynaptic neurons for all the *i*-th original classes remain inactive (*y*_*i*_ = 0), given the training image is identified as a cat. According to the general form of the Hebbian rule (Dayan and Abbott, 2001), the change in the weight vector is expressed as Δ***w***_cat_∝***x***·*y*_cat_, resulting in ***w***_cat_ = Δ***w***_cat_∝***x***. Conversely, Δ***w***_*i*_ = 0 for the other classes since *y*_*i*_ = 0. Therefore, the synaptic connections between the active presynaptic neurons and the postsynaptic neuron corresponding to the cat are strengthened, in line with Hebbian theory.

While Hebbian learning may not occur instantaneously in the classical framework of animal brains (Hebb, 1949), it has been shown to actually occur with a small number of spikes (Froemke et al., 2006). In the context of one-shot learning in the brain, it is realistic to assume that multiple spikes could occur in a one-shot event rather than just a single spike. Therefore, incorporating such fast Hebbian processes in one-shot learning scenarios may align with reality.

### 2.3 Our weight imprinting: direct one-shot learning

Qi's method, which can be interpreted as incorporating a Hebbian-like process as described above, focuses solely on cosine similarity. This focus results in changes to the weights of the original backbone DNN and the elimination of its bias. In contrast, we propose a method named DONE, which incorporates only a single Hebbian-like process, by emulating a brain neural process.

In Figure 1A, considering the distinct dimensions/scales of neural activity and synaptic weight in both real brains (as physical constraints of neurons) and ANNs, directly using ***x*** as the new ***w***_cat_ is not realistic. Therefore, converting the statistical properties of neural activity into those of synaptic weight is necessary: ***w***_cat_ = *F*_***x***→***w***_(***x***·*y*_cat_). The statistical properties of the new ***w***_cat_ vector should align with those of synaptic weights, i.e., the original weight matrix ***W***_ori_. Thus, the transformation function *F*_***x***→***w***_ must incorporate the information of ***W***_ori_, leading to ***w***_cat_ = *F*(***x***, ***W***_ori_). This approach computationally represents the inherent physical constraints of synapses, regardless of the brain's awareness of such information about ***W***_ori_.

The procedure of DONE, as shown in Figure 1, consists solely of adding a new-class weight vector ***w***_*j*_ and a bias scalar *b*_*j*_ as follows:


(2) $$\begin{array}{l} w_{j}=F\left(x_{\text{new}},W_{\text{ori}}\right), \end{array}$$

(3) $$\begin{array}{l} b_{j}={\tilde{b}}_{\text{ori}}, \end{array}$$

where *F*(***x***_new_, ***W***_ori_) normalizes the final-dense-layer input from the new-class image ***x***_new_ using the information of original weights (***W***_ori_) as the reference distribution. Since information about the bias value for the new class *b*_*j*_ is not obtained from the input, the median of the original bias vector ${\tilde{b}}_{\text{ori}}$ is adopted as the bias value. Then, it is done.

For the normalization in DONE (in Equation 2), we employed four types of functions to ensure that: (I) the mean values (i.e., the 1st central moment) of both the new and original weights are identical, (II) the variance values (the 2nd central moment) are identical, (III) both the mean and variance values are identical, and (IV) all statistical properties are identical. These four variations are referred to as DONE(I) through DONE(IV).

In considering the brain, a function that results in new weights having statistical properties more similar to those of the original weights is likely to be more realistic. This is because such a function better represents the physical characteristics of synaptic connections. Therefore, the type (IV) function should be the most suitable in this respect.

For applications, it is uncertain whether only the 1st and/or 2nd central moments are sufficient, especially in situations where the 3rd or higher central moments might differ. One of the most straightforward solutions for any situation is to non-parametrically ensure all statistical properties are identical. As explained in the next subsection, the type (IV) function can be implemented using quantile normalization, a non-parametric method. Thus, the type (IV) is one of the methods that does not require any special assumptions for its application and suitable for a wide range of scenarios.

Therefore, in this study, we employed DONE(IV) as the standard method, and unless otherwise specified, the term “DONE” refers exclusively to DONE(IV). In line with the non-parametric approach utilized for the normalization function, we used the median value as the central tendency for the original bias vector (in Equation 3).

### 2.4 Quantile normalization to better reflect real neural mechanisms

Neural activity and synaptic weight differ in dimensions and scales, and their relationships are typically non-linear in both real brains and ANNs. For instance, Figure 1B—(i) and (ii) illustrate the frequency distributions of neural activity in ***x*** and synaptic weight in ***w***_*i*_, respectively, showing distinct shapes in an actual DNN. This discrepancy indicates that the general form of the Hebbian process, which is often restricted to linear interactions, cannot adequately capture these non-linear relationships.

We implemented the non-linear relationship using quantile normalization (Amaratunga and Cabrera, 2001; Bolstad et al., 2003), ensuring that the frequency distribution of the new weight vector ***w***_*j*_ aligns with that of the multiset of typical elements of the original ***w***_*i*_ vectors ($W_{\text{typical}}$), as shown in Figure 1B—(iii). Quantile normalization, a straightforward and standard technique in bioinformatics, effectively facilitates this non-linear scale transformation.

In quantile normalization, each element value in the resulting vector ***w***_*j*_ is matched with the corresponding value in the reference multiset $W_{\text{typical}}$. Specifically, we start by transforming the value of the most active (1st) neuron in ***x***_new_ to the highest (1st) weight value in $W_{\text{typical}}$. This process is then repeated sequentially for the 2nd, 3rd, ⋯ , and *M*-th most active neurons. As a result, while the ranking of each neuron in ***x***_new_ is preserved, the value assigned to each rank becomes identical to that in $W_{\text{typical}}$. The resultant vector produced through this process is ***w***_*j*_. Consequently, all statistical properties of the elements in ***w***_*j*_ become identical to those in $W_{\text{typical}}$, i.e., their frequency distributions are the same.

For $W_{\text{typical}}$, to represent the concept of physical constraints in synaptic connections, we utilized all *N*×*M* elements of the flattened ***W***_ori_. These elements were divided into *M* groups based on their ranking, and then, the median value of each group of *N* elements was calculated to form an *M*-element multiset $W_{\text{typical}}$. For instance, in a DNN model like ViT-B/32 (*N* = 1, 000, *M* = 768), the highest value in $W_{\text{typical}}$ is the median of the top 1st to 1,000th elements among the 768,000 elements of ***W***_ori_, while the lowest value is the median of the 767,001st to 768,000th elements (refer to Supplementary Figure S1 for a comparison with another method to construct the reference multiset).

Note that the use of quantile normalization in this process deviates from the traditional Hebbian form. While the term “Hebbian” is useful for easily conveying its relationship to brain processes, the key aspect is not strictly its adherence to a Hebbian process but rather its representation of a process similar to that of the brain. The Hebbian principle, broadly encapsulating the concept that simultaneous activation of neurons strengthens their synaptic connection, as in a phrase like “neurons that fire together wire together” (Hebb, 1949), is often restricted to a linear form in computational neuroscience for precise definition (Dayan and Abbott, 2001).

According to the precise definition, DONE(I) to DONE(III) are based on a single Hebbian process. Strictly speaking, DONE(IV) does not conform to a traditional Hebbian process. However, we consider DONE(IV) as a Hebbian-like process, which incorporates quantile normalization in line with Hebbian theory for rapid processing. This approach is likely more aligned with reality than the traditional form of the Hebbian process, particularly in the context of modeling a fast Hebbian process through weight imprinting. Regardless, as shown below, one-shot learning without modifying the backbone is feasible using either a single Hebbian or Hebbian-like process.

### 2.5 Limitations, applications, and negative impacts of DONE

An inherent limitation of DONE is that it requires an ANN model with a dense layer for classification, as previously mentioned. Despite this, DONE is versatile and can be utilized in a wide range of applications involving DNN classifiers, including out-of-distribution detection (Yang et al., 2021). However, there are various potential negative societal impacts associated with such broad applications. For example, immoral classification or discrimination may occur when classifying human-related data, such as facial images, voices, and personal features. It is important to note that while DONE is a weight imprinting method, it does not involve modifications to the backbone models, unlike Qi's method. Furthermore, the new-class weight vectors created by DONE might be less distinguishable from the original weight vectors based on their statistical characteristics, potentially increasing the risk of secretive modifications.

## 3 Materials and methods

### 3.1 Backbone DNN models

In this study, we utilized several backbone models, including ViT-B/32 (Dosovitskiy et al., 2020) with “vit-keras” (Morales, 2020), EfficientNet-B0 (Tan and Le, 2019) with “EfficientNet Keras (and TensorFlow Keras)” (Iakubovskii, 2019), InceptionV1 (Szegedy et al., 2015) [as used in (Qi et al., 2018)] with “Trained image classification models for Keras” (Andrews, 2017), ResNet-12 (He et al., 2016) with “tf2cv” (Semery, 2018), ResNet-50 (He et al., 2016), MobileNetV2 (Sandler et al., 2018), and VGG-16 (Simonyan and Zisserman, 2015) with TensorFlow (Abadi et al., 2015). All these models were pre-trained on ImageNet.

### 3.2 Image datasets

We used CIFAR-100 and CIFAR-10 (Krizhevsky and Hinton, 2009) for additional class data with TensorFlow (Abadi et al., 2015). For transfer learning, we used CIFAR-FS (Bertinetto et al., 2018) with Torchmeta (Deleu et al., 2019). The performance of the models was tested using ImageNet (ILSVRC2012) images (Deng et al., 2009; Russakovsky et al., 2015). We utilized 67 categorizations (Eshed, 2020) of ImageNet 1000 classes for a coarse 10 categorization, as shown in **Figure 5A**.

## 4 Results and discussion

### 4.1 One-class addition by one-shot learning

**Figure 2:**
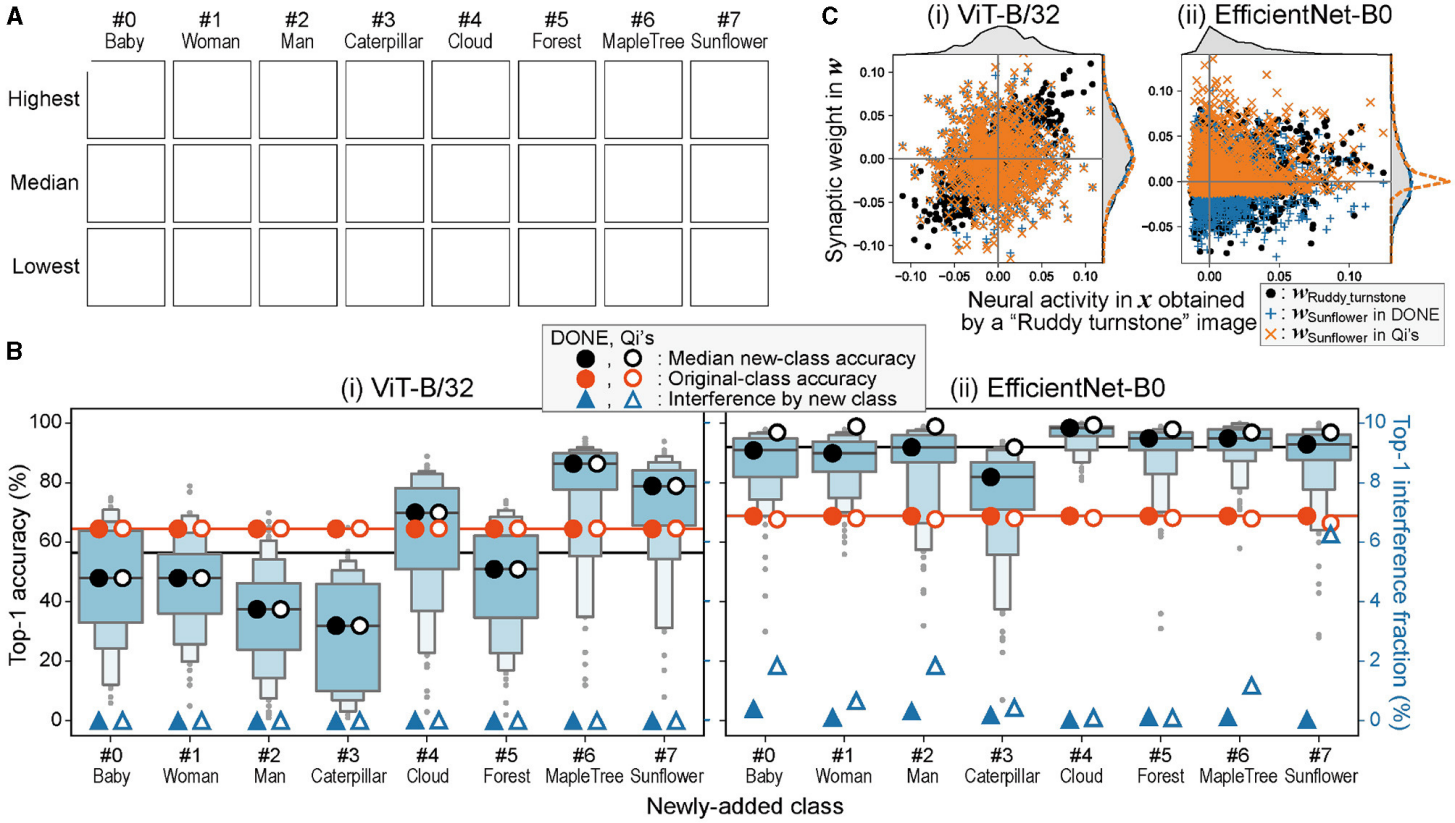
One-class addition by one-shot learning. **(A)** Representative images of the newly-added CIFAR-100 classes (Due to copyright issues, the original images in this figure have been removed. Images available from the corresponding author upon request). Each image was chosen as a representative because the model that learned the image showed the highest, median, and lowest accuracy in each class in **(B)**—(i). **(B)** Letter-value plots of top-1 accuracy of the one-class-added models obtained by one-shot learning with DONE in classification of new-class images. The median top-1 accuracy of the new-class classification (black circles), top-1 accuracy in original-class classification (orange circles), and the fraction of the interference with the original-class classification by the newly-added class (blue triangles) are plotted for DONE (closed) and Qi's method (open). Black and orange lines show the mean of the eight closed circles. **(C)** The relationship between ***x*** and ***w*** vectors when an image of “Ruddy turnstone” is input and it is miss-classified as “Sunflower” only in the case of Qi's method with EfficientNet-B0. The frequency distributions of elements of each vector are also shown outside the plot frames.

Initially, in line with our motivation, we evaluated the performance of our method, DONE, when adding a single image from a new class to a DNN model pre-trained with ImageNet's 1,000 classes. It is important to note that in this study, “DONE” specifically refers to “DONE(IV) using quantile normalization,” unless stated otherwise. For backbone DNN models, we primarily employed ViT-B/32 and EfficientNet-B0 as representatives of vision transformer and CNN, respectively. As new additional classes, eight distinct classes, “baby,” “woman,” “man,” “caterpillar,” “cloud,” “forest,” “maple_tree,” and “sunflower,” were selected from CIFAR-100, which are not present in ImageNet (as shown in Figure 2A). The weight parameters for each additional class, ***w***_*j*_, were generated from a single image, thus expanding the model to 1,001 classes. To enable stochastic evaluations, 100 different models were constructed, each using a different training image for every additional class.

Figure 2B shows the letter-value plots of accuracy for each additional class (chance level 1/1, 001). The mean of the median top-1 accuracy of the eight classes using DONE was 56.5 and 92.1% for ViT-B/32 and EfficientNet-B0, respectively (black line). When the mean accuracy was compared with the accuracy of the ImageNet validation test by the original 1000-class model (orange line: 65 and 69%), the mean with ViT-B/32 showed no significant difference, and the mean of EfficientNet-B0 was significantly greater (one-sample *t*-test; two-sided with α = 0.05, in all statistical tests in this study). The higher accuracy than the original classes in EfficientNet-B0 is strange, and it is considered that EfficientNet-B0 tends to classify any image belonging to the new class (see below). Thus, the strangely high accuracy does not indicate good performance but rather reflects a potential drawback in interfering with the original image classification.

An obvious fact in one-shot learning is that a bad training image produces a bad performance, for example, the lowest accuracy was 6% in ViT-B/32 when the training image was a baby image, as shown at the bottom left in Figure 2A. However, in practice, a user is supposed to use a representative image for training. Therefore, we believe that the low performance owing to a bad training image is not a significant issue.

We investigated the interference of class addition on the classification performance of the original 1,000 classes. We evaluated the original 1000-class model and eight 1,001-class models that showed the median accuracy using all 50,000 ImageNet validation images (Figure 2B). The difference between the accuracy of the original 1000-class model (orange line) and the mean accuracy of the eight 1,001-class models (orange closed circles) was < 1% (0.004 and 0.664% for ViT-B/32 and EfficientNet-B0, respectively).

Figure 2B also shows the fraction of ImageNet validation images in which the output top-1 answer of the added model was the new class (thus incorrect) in 50,000 images (blue closed triangles; right axis). This interference fraction was low in ViT-B/32, and for example, only two images out of 50,000 were classified as “baby.” When we checked the two images, both images indeed contained a baby, although its class in ImageNet was “Bathtub.” Therefore, the observed interference in ViT-B/32 was not a mistake but just the result of another classification. EfficientNet-B0 showed a significantly greater fraction of interference than ViT-B/32 (Wilcoxon signed-rank test), but we also confirmed that a similar phenomenon occurred, for example, 198 of the 204 ImageNet-validation images classified as “baby” in EfficientNet-B0 contained humans or dolls.

We also compared DONE with Qi's method. Open circles and triangles show the results obtained using Qi's method instead of DONE in the same tests described above. When the backbone model was EfficientNet-B0, the strangely high accuracy (paired sample *t*-test) and interference fraction (Wilcoxon signed-rank test) were significantly greater by Qi's method than by DONE. In addition, a significant outlier of decreased accuracy in the ImageNet validation test was observed (orange open circle for “Sunflower”; Smirnov–Grubbs test). However, these differences were not significant for ViT-B/32.

To investigate the cause of the difference between DONE and Qi's method, especially regarding the greater interference by Qi's method in EfficientNet-B0, we plotted ***w***_Sunflower_ and ***w***_Ruddy_turnstone_ against ***x*** obtained from an image of “Ruddy turnstone” (Figure 2C ). Note that all the vectors here are *L*_2_-normalized for comparison, and thus, DONE and Qi's methods have a common ***w***_Ruddy_turnstone_ and ***x***. In the case of ViT-B/32, the shapes of the frequency distributions of all these vectors are similar, and ***w***_Sunflower_ values of DONE and Qi's method are similar.

On the other hand, in EfficientNet-B0, the shapes of the frequency distributions are more different between ***w***_Ruddy_turnstone_ and ***x*** than ViT-B/32, and thus, the shapes of frequency distributions are more different between ***w***_Ruddy_turnstone_ and ***w***_Sunflower_ by Qi's method than by DONE. Then, by Qi's method, ***x*** is more similar to ***w***_Sunflower_ than ***w***_Ruddy_turnstone_ because it is not neural match, but the statistical properties are similar, that is, Qi's method with EfficientNet-B0 tends to classify every image into a new class. This is the basis of the problem owing to the neglect of the differences in statistical properties between neural activity and synaptic weight. Therefore, the difference between DONE and Qi's method appears in the interference when the statistical properties of ***x*** and ***w***_*i*_ vectors in the backbone DNN are different (thus, the results in ViT-B/32 are similar between DONE and Qi's method).

### 4.2 Multi-class addition and K-shot learning

DONE was able to add a new class as above, but it might just be because the models recognized the new-class images as out-of-distribution data, that is, something else. Therefore, it is necessary to add multiple new similar classes and check their classification. In addition, it is necessary to confirm whether the accuracy increases by increasing the number of training images because in practical uses, users will prepare not just one training image but multiple training images for each class.

**Figure 3:**
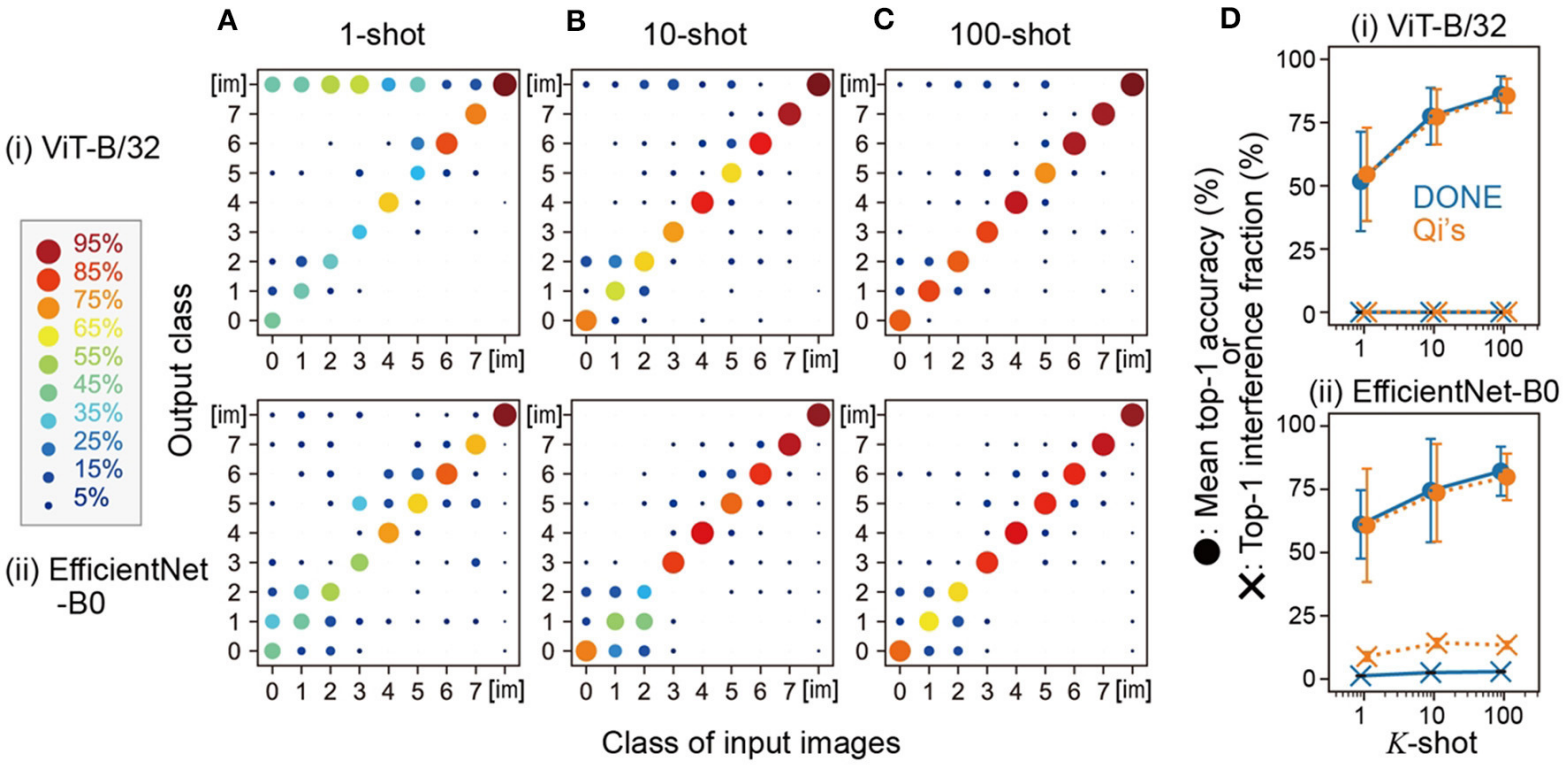
Multi-class addition and *K*-shot learning. **(A–C)** show the results of the 1008-class model constructed by 1, 10, and 100-shot learning, respectively. The horizontal and vertical axes represent the class of the input images and the output class, respectively, with class numbers as shown in Figure 2A. The class [im] comprises the 1,000 classes of ImageNet. **(D)** Summary of the mean accuracy and the interference with original-class classification by DONE (blue symbols) and Qi's method (orange symbols). Error bars represent the standard deviation of the eight classes.

Specifically, we used one image from each of the eight classes and added eight new classes to the original 1,000 classes using DONE for one-shot learning. We evaluated this 1008-class model using 100 CIFAR test images for each of the eight classes and 10,000 ImageNet validation images. Figure 3A shows the results of the output of the representative model constructed by one-shot learning, in which one image that showed the median accuracy in Figure 2B was used as a standard training image for each class. In both backbone DNNs, the fraction of output of the correct class was the highest among the 1,008 classes, and the mean top-1 accuracies of the eight classes were 51.8 and 61.1% in ViT-B/32 and EfficientNet-B0, respectively. Therefore, DONE was also able to classify newly added similar classes together with the original classes in both DNNs.

Next, we increased the number of training images for the *K*-shot learning. In the case of 10-shot learning (Figure 3B), each of the 10 images was input to obtain each ***x***, and the mean vector of the 10 ***x*** vectors was converted into ***w***_*j*_, according to Qi's method. For this representative 10-shot model, we used 10 images whose index in CIFAR-100 was from the front to the 10th in each class. We also tested 100-shot learning in the same manner (Figure 3C). We found that such a simple averaging operation steadily improved accuracy (Figure 3D summarizes the mean accuracy).

When we used Qi's method, compared with the case of DONE, ImageNet images were significantly more often categorized to the new classes as interference when the backbone model was EfficientNet-B0 (paired sample *t*-test), whereas there was no significant difference in the mean accuracy between DONE and Qi's method with both backbone DNNs (Figure 3D). In any case, both DONE and Qi's methods were able to perform one-shot learning that was not simply out-of-distribution detection, with a similar degree of accuracy. However, in interference, there appears to be differences both in weight imprinting methods and backbone DNN models.

### 4.3 Comparative analysis of various backbone DNNs and weight imprinting methods

We compared the accuracy and interference of the one-shot multi-class addition task using various well-known backbone DNN models and weight imprinting methods. The task was the same as that shown in Figure 3, and the chance level was 1/1, 008.

**Figure 4:**
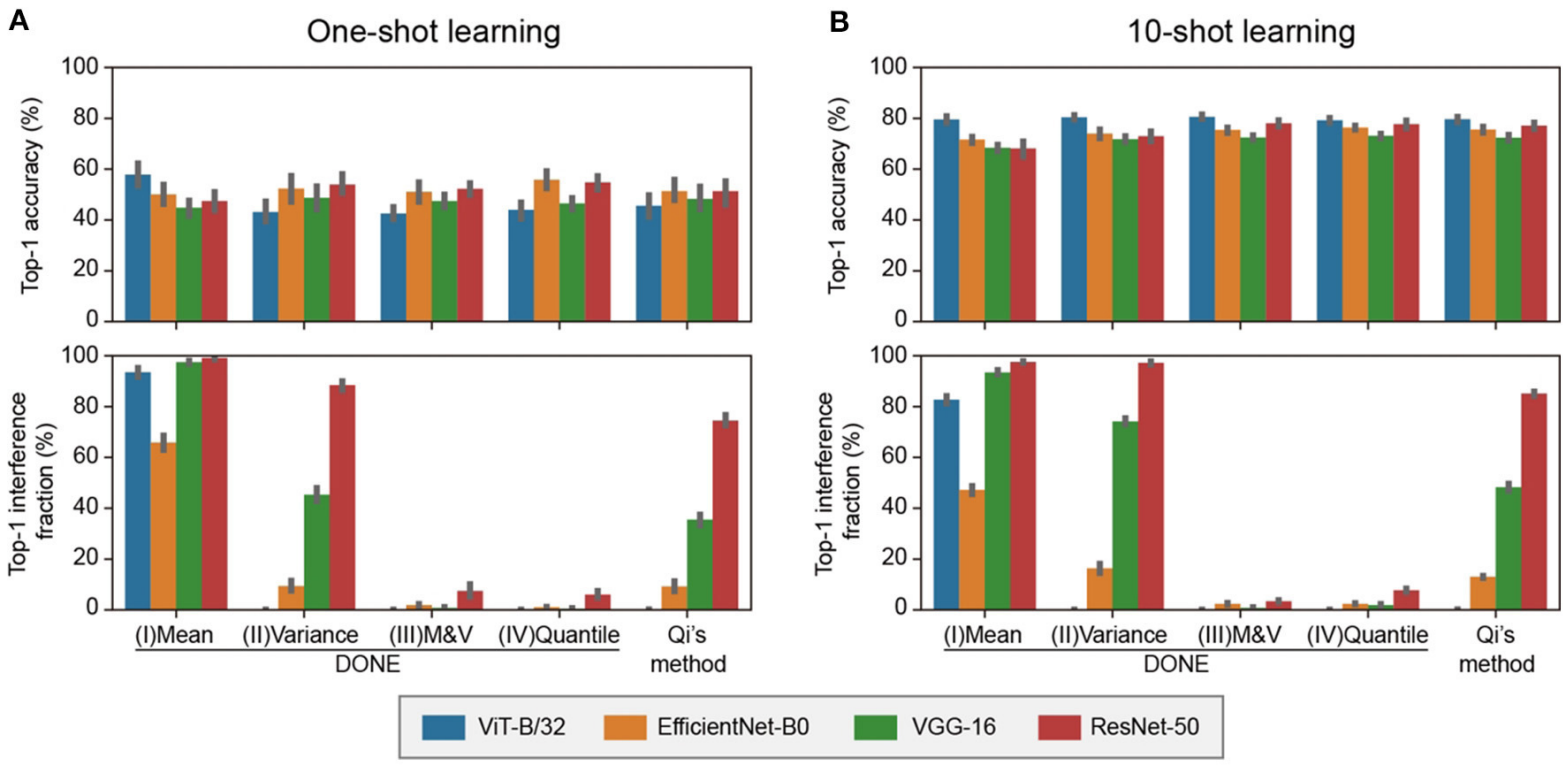
Comparative analysis of various backbone DNNs and weight imprinting methods in 1-shot and 10-shot 8 class addition tasks. **(A)** 1-shot class addition. For each of the eight classes, one training image was randomly selected. This procedure was replicated 10 times, and the mean value is displayed in a bar graph. Error bars represent the 95% confidence intervals. **(B)** 10-shot class addition. This test was conducted similarly to the one-shot class addition task, with 10 images per class used for training.

Figure 4A shows the results of the one-shot learning. In accuracy, there was no statistically significant difference between all weight imprinting methods (Dwass-Steel-Critchlow-Fligner test). As a representative, with DONE(IV), the accuracy was 44, 59, 47, and 55% for ViT-B/32, EfficientNet-B0, VGG-16, and ResNet-50, respectively. These values, averaged over the four DNNs, were ~80% of the accuracy of ImageNet validation test by the original 1000-class model (65, 69, 60, and 62% for ViT-B/32, EfficientNet-B0, VGG-16, and ResNet-50, respectively).

Regarding interference, the profiles for the four backbone DNNs were different depending on the weight imprinting methods. There was no statistically significant difference between DONE(III) and DONE(IV) and between DONE(II) and Qi's method, and there were significant differences between all the other combinations (Dwass-Steel-Critchlow-Fligner test). The difference between DONE(I), (II), and (III) showed that the normalization with both the mean and variance, i.e., 1st and 2nd central moments, was required to avoid the large interference. The similarity between DONE(III) and DONE(IV) suggested that the normalization with 3rd or higher central moments did not provide a significant effect on the interference, at least in this case. The results of 10-shot learning showed a similar trend as 1-shot learning, but the accuracy values were greater (Figure 4B).

For practical applications, DONE(III) and DONE(IV) each have opposite advantages. Since the normalization of DONE(III) is linear, the new ***w***_*j*_ distribution retains the shape of the distribution of ***x***. This method would not require any special assumptions as it utilizes the distribution of ***x*** directly. Conversely, in DONE(IV), the normalization is non-linear, and the new ***w***_*j*_ distribution non-parametrically retains the shape of the original ***W***_ori_ distributions. This method also does not require special assumptions, as it employs the original distribution of ***W***_ori_ non-parametrically. Therefore, in actual applications, whether to use DONE(III) or DONE(IV) depends on whether the distribution of ***x*** or ***W***_ori_ is more suitable for the new ***w***_*j*_ vector, respectively.

The interference observed with Qi's method was substantial enough to pose a severe problem in practical applications. For example, in ResNet-50, most (75%) of the original-class ImageNet images were misclassified into new classes. This severe interference might explain why the previous weight imprinting method has not been widely adopted, despite its promising properties for one-shot class addition. DONE(III) and DONE(IV) significantly reduced interference to less than one-tenth (7.4 and 6.0%, respectively).

It is understandable that there was no significant difference between DONE(II) and Qi's method. Qi's method uses *L*_2_ normalization for both ***x*** and all ***w*** vectors. Thus, all new ***w***_*j*_ vectors and original ***w***_*i*_ vectors have the identical 2nd moments. DONE(II) normalizes new ***w***_*j*_ vectors so that the 2nd central moments are the same as the original ***W***_ori_ matrix. Therefore, both methods have normalized the scale but not the average value.

Focusing on the metric of cosine similarity, it is understandable that normalization of the average value should be ignored. However, in the case of weight imprinting, normalization of the average value is important, as well as the scales. For example, consider an extreme case where all values of the ***x*** vector are positive, and the values of ***w*** vectors include positive and negative values with the mean value of zero. If we adopt the ***x*** vector as a new ***w***_*j*_ vector without normalizing the average value, all values of this new ***w***_*j*_ vector become positive. All values of Hadamard product ***x***°***w***_*j*_ become positive, and the dot product ***x***·***w***_*j*_ and the cosine similarity, when L2 normalized, would tend to become a larger value for the new class than the other original classes. As a result, without normalization with the average value, the interference with the original classes by the new class tends to be large when the average values are different between ***x*** and ***w***_*i*_ vectors.

The cause of this interference is considered to be the difference in the shapes of the frequency distributions of ***x*** and ***w***_*i*_. As far as we tested (Supplementary Figure S2), in all two vision transformers and six CNNs, ***w***_*i*_ had a bell-shaped distribution with an average value of approximately zero. On the other hand, for ***x***, two vision transformers had also bell-shaped distributions with average values of approximately zero, while all six CNNs had right-tailed distributions. In particular, among the three CNNs tested in Figure 4, EfficientNet-B0 had negative values in ***x*** vectors with a small positive average value, but the values of ***x*** vectors in VGG-16 and ResNet-50 were all greater than or equal to zero, which was consistent with the above explanation.

Although it is not clear why only the distribution of ***x*** in vision transformers is bell-shaped, vision transformers are known to uniformly acquire information from the entire input through patch division and self-attention, integrating extensive input information and maintaining more uniform representations across all layers (Dosovitskiy et al., 2020; Naseer et al., 2021; Raghu et al., 2021). These characteristics are different from CNNs, where specific neurons tend to respond strongly to specific local patterns due to convolution, leading to more robust features against inputs in vision transformers (Cordonnier et al., 2020; Fort et al., 2021). Such difference between locality and extensive coverage would be related to the difference between right-tailed and bell-shaped distributions of ***x***.

The bell-shaped distributions of the neural activity ***x*** in vision transformers do not deviate from the situation in the brain. In ANNs, values representing neural activity are often zero or positive numbers to represent the spikes. It would be a natural assumption that the frequency distribution is right-tailed for the neural activity. However, at least ***x*** here is the value of the final-dense-layer input of DNNs. The final-dense-layer input can be considered to correspond to the higher visual cortex in the brain (Tsunoda et al., 2001; Yamins et al., 2014). Therefore, rather than the activity of each neuron, it is considered to represent the activity of a cluster made up of multiple neurons. Therefore, from the central limit theorem, it is not inconsistent with the situation in the brain that the distribution is expressed more like a normal distribution than a right-tailed distribution.

### 4.4 Principal component analysis of weight vectors

Qi's method showed greater interference in the classification of original-class images than DONE when the backbone DNN was EfficientNet-B0. Moreover, even with DONE, EfficientNet-B0 showed greater interference than ViT-B/32 and strangely high accuracy in the 1,001-class model, even though DONE did not change the weights for the original classes and transformed the new-class weights so that the statistical properties were the same as those of the original-class weights. Therefore, there should be at least two reasons for the results observed with EfficientNet-B0.

**Figure 5:**
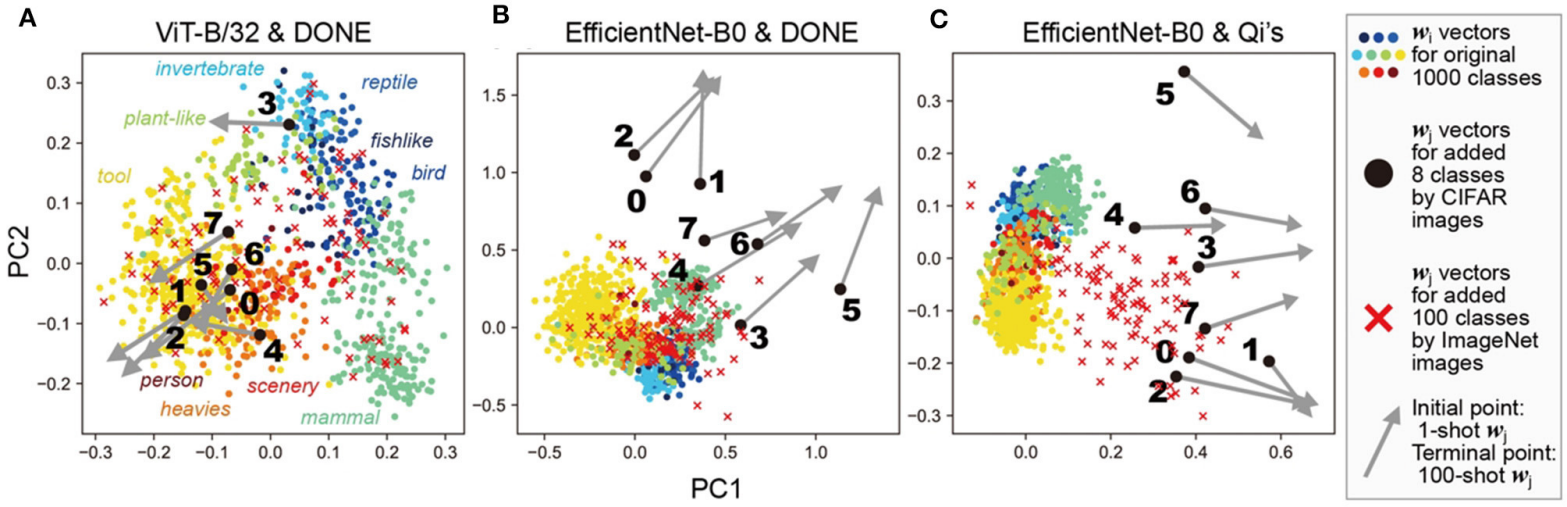
Principal component analysis of weight vectors. This figure shows PCA of each ***w***_*i*_ and ***w***_*j*_ vector in the one-shot 1008-class models as shown in Figure 3A. Different colors represent a coarse 10 categorization of the original classes for ***w***_*i*_. Additionally, 100 ***w***_*j*_ vectors, obtained by inputting 100 different ImageNet images, are also shown to illustrate the distribution of new-class vectors. The DNN models and weight imprinting methods used were: **(A)** ViT-B/32 and DONE, **(B)** EfficientNet-B0 and DONE, **(C)** EfficientNet-B0 and Qi's method.

To investigate these reasons, we analyzed the ***W*** matrix (***w***_*i*_ and ***w***_*j*_ vectors) of the one-shot 1008-class models, as shown in Figure 3A (and the corresponding models by Qi's method), using principal component analysis (PCA; Figure 5).

In ViT-B/32 with DONE (Figure 5A, Qi's methods showed similar results, see Supplementary Figure S3); newly added ***w***_*j*_ vectors (black circles, with the ID number of newly-added eight classes) were comparable to those of the original classes ***w***_*i*_ (colored circles). For example, ***w***_*j*_ vector of a new class “caterpillar (3 in Figure 5A)” was near ***w***_*i*_ of original “invertebrate” classes. In addition, even when we obtained ***w***_*j*_ by inputting ImageNet images (red crosses; validation ID from the front to the 100th), the ImageNet ***w***_*j*_ vectors were distributed within a similar range.

In EfficientNet-B0 with DONE (Figure 5B), most of the newly-added 8-class ***w***_*j*_ (black circles) were out of the distribution (meaning out of minimal bounding ellipsoid) of ***w***_*i*_ of the original 1,000 classes. On the other hand, most of the ImageNet ***w***_*j*_ (red crosses) were inside the distribution of ***w***_*i*_. Similar results were obtained with DONE(III) (Supplementary Figure S3). Therefore, in the case of DONE, the main reason for the observed interference and the strangely high accuracy in EfficientNet-B0 compared with ViT-B/32 is considered to be the difference between the characteristics of image data in ImageNet and CIFAR datasets. These results are consistent with known facts that ViT-B/32 is considered to be better at predictive uncertainty estimation (Guo et al., 2017; Minderer et al., 2021), more robust to input perturbations (Bhojanapalli et al., 2021), and more suitable for out-of-distribution detections (Fort et al., 2021) than CNNs.

In EfficientNet-B0 with Qi's method (Figure 5C), most of the 8-class ***w***_*j*_ (black circles) and ImageNet ***w***_*j*_ (red crosses) were out of the distribution of ***w***_*i*_ of the original 1,000 classes. The difference in the distributions between the original ***w***_*i*_ and ImageNet ***w***_*j*_ was considered to indicate the difference in the mean values of ***x*** and ***w***_*i*_ vectors in EfficientNet-B0.

In the case of 100-shot learning (the terminal points of the gray arrows, as shown in Figure 5), ***w***_*j*_ moved away from the cluster of the original ***w***_*i*_ in all three cases, although their performance was better than that of the 1-shot learning. Therefore, 100-shot ***w***_*j*_ vectors were considered to work somehow in a different way from the original ***w***_*i*_ vectors.

### 4.5 Few-shot learning in transfer learning context

**Figure 6:**
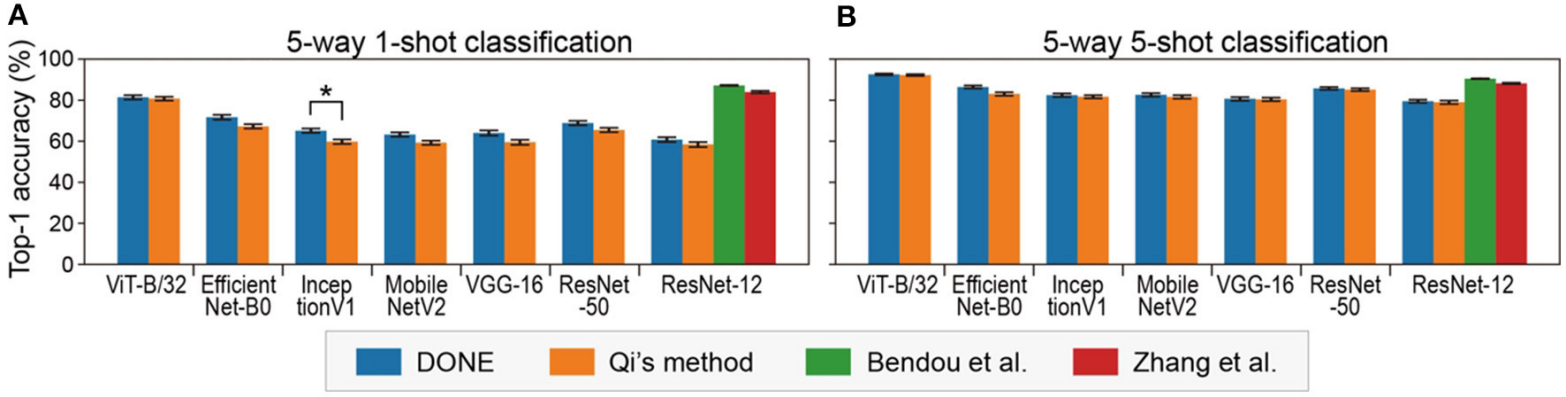
Five-way 1-shot **(A)** and 5-shot **(B)** classification accuracy on CIFAR-FS with various backbone DNNs. Error bars indicate the standard error. Asterisks indicate significant differences between DONE and Qi's method (Dwass-Steel-Critchlow-Fligner test).

In terms of practical application, DONE is a method for few-shot class addition tasks, not for few-shot transfer learning. However, transfer learning with DONE is convenient for the evaluation of DNNs because the performance is uniquely determined by the backbone DNN without any randomness. We examined the 5-way (five classes) 1-shot task of CIFAR-FS, which is a type of standard task in 1-shot classification. Specifically, we used each single image in five out of 100 classes of CIFAR-100 to construct a model and evaluated the model using 15 images in each class. The combination of the five classes (and the corresponding training images) was randomly changed 100 times (Figure 6A). In addition, 5-way 5-shot tasks were tested in a similar manner (Figure 6B).

We found that ViT-B/32 significantly outperformed the other DNNs under all conditions (Dwass-Steel-Critchlow-Fligner test). In class addition tasks, there are at least two important performance indexes, accuracy and interference, but in transfer learning, only accuracy is evaluated. In this sense, it might be suitable for directly comparing the performance of backbone DNNs models.

There was not much difference between the DONE and Qi's methods, but DONE was significantly better than Qi's method with a CNN model (InceptionV1) and was never significantly worse. The reason for this small difference is not clear, but since the differences between two methods here are only in the weight distribution, the bell shape might be better than the right-tailed for the weight distribution. This is consistent with the fact that the weight distribution was bell-shaped in all DNNs, including vision transformers and CNNs (Supplementary Figure S2).

Figure 6 also clearly shows how much other state-of-the-art 1-shot learning methods, with additional optimization (methods in the study by Bendou et al., 2022; Zhang et al., 2022), outperform DONE, as the baseline without optimization, in the same test with a common backbone DNN (ResNet-12). Basically, the performance of DONE should be at the bottom in transfer learning, and transfer learning is not a practical application task for DONE (the accuracy was similar to SDG, stochastic gradient descent, one of the simplest optimizers; Supplementary Figure S4). However, it may be used as a baseline for few-shot learning methods in transfer learning or learning from scratch, i.e., to quantify how much better than DONE because DONE does not include any randomness.

## 5 Conclusion

In this study, we investigated a brain-inspired method for one-shot class addition tasks in vision to understand the brain's 1-shot learning and develop a practical application method. We introduced a novel Hebbian interpretation for weight imprinting methods and demonstrated that a single fast Hebbian/Hebbian-like process can enable pre-trained DNNs to perform 1-shot class addition without any modification to the backbone DNNs. The accuracy of the newly added eight classes was ~80% of the accuracy of the original 1,000 classes in a 1008-way classification task, on average of using well-known DNNs: ViT-B/32, EfficientNet-B0, VGG-16, and ResNet-50. Our weight imprinting method, DONE(III) with linear normalization for 1st and 2nd central moments and DONE(IV) with non-linear non-parametric quantile normalization, significantly decreased the severe interference observed in the previous Qi's method. For example, Qi's method misclassified 75% of original-class images into newly added classes with ResNet-50, DONE(III), and DONE(IV) that reduced the interference to less than one-tenth. The primary cause of the interference was identified as the difference in the statistical characteristics between neural activity and synaptic weight, which was evident in CNNs but not in vision transformers. The advantages of DONE as a practical application method over Qi's weight imprinting method are (i) a simpler procedure, (ii) no backbone modification, and (iii) minimal interference with original classification. Moreover, DONE as a weight imprinting method offers (iv) no need for optimization, parameters, hyperparameters, or randomness, making it replicable for any user. As the performance of DONE is entirely dependent on the backbone DNNs, and with the ongoing development of DNNs, the range of tasks achievable with DONE in practice will continue to grow. Furthermore, in the current situation where fast Hebbian plasticity is a hot topic in neuroscience, demonstrating that a single fast Hebbian/Hebbian-like process can enable 1-shot learning in DNNs in this study which would be a significant contribution to understanding brain 1-shot learning.

## Data availability statement

Publicly available datasets were analyzed in this study. This data can be found here: CIFAR (https://www.cs.toronto.edu/~kriz/cifar.html). Most of the results in this study are based on ImageNet, which does not own the copyright of the images. Therefore, we have uploaded the core part of the code for our method, excluding the ImageNet data, to the following URL on GitHub (hosodakazufumi_tfdone_2022): [https://github.com/hosodakazufumi/tfdone].

## Author contributions

KH: Conceptualization, Data curation, Formal analysis, Funding acquisition, Investigation, Methodology, Project administration, Resources, Software, Supervision, Validation, Visualization, Writing—original draft, Writing—review & editing. KN: Data curation, Formal analysis, Methodology, Writing—review & editing. SS: Conceptualization, Supervision, Writing—review & editing. TM: Conceptualization, Supervision, Writing—review & editing. HK: Conceptualization, Project administration, Supervision, Writing—review & editing. IO: Conceptualization, Investigation, Project administration, Supervision, Writing—review & editing.
