# Supplementary material for: A single fast Hebbian-like process enabling one-shot class addition in deep neural networks without backbone modification
Source: Front Neurosci. 2024 Jun 12;18:1344114. doi: 10.3389/fnins.2024.1344114 (PMC11202076; doi:10.3389/fnins.2024.1344114)
Supplement: Supplementary file 1 [file Data_Sheet_1.PDF]

Supplementary Material

A Single Fast Hebbian-like Process Enabling One-Shot Class Addition in Deep Neural Networks

Without Backbone Modification

This file includes

- Figures S1
- Figures S2
- Figures S3
- Figures S4

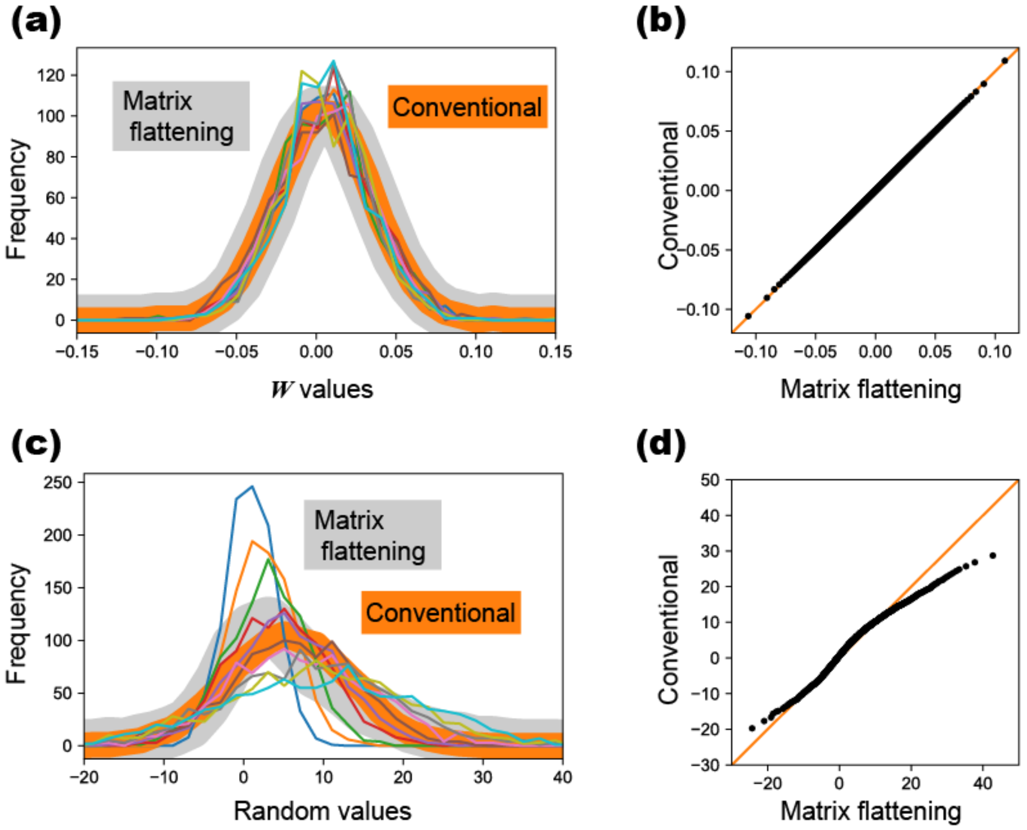

Figure S1. Comparison of methods for obtaining the reference multiset ($\mathcal{W}_{\text{typical}}$), as a typical set of elements of weights for original classes. We flattened the original weight matrix (***W***) in the paper, as shown in the main text in order to implement the concept of physical constraints of neurons (synaptic weights). This averaging method is different from a conventional method mainly used in Bioinformatics such as for DNA microarray data (Amaratunga and Cabrera 2001; Bolstad et al. 2003), which uses the mean of each rank of vectors in the matrix for the average value of each rank of $\mathcal{W}_{\text{typical}}$. (a) Frequency distribution of $\mathcal{W}_{\text{typical}}$ obtained by the matrix-flattening method (gray-bold line) and the conventional method (orange-bold line), with 10 example $\boldsymbol{w}_i$ vectors (thin lines), of ViT-B/32. (b) Relationship between the two averaging methods. The results show that there is little difference between these methods, because 1000 $\boldsymbol{w}_i$ vectors are similar. We are not claiming that the matrix-flattening method is better than the conventional method, because which method is practically better depends on the situation. In principle, the conventional method keeps the mean of the matrix exactly, while the matrix-flattening method approximately keeps the median. The width of averaged distribution by the conventional method represents the average width of the distributions of the respective vectors, while that by the matrix-flattening method represents the width of the distribution of the original matrix ***W***. Thus, the conventional method produces a narrower distribution than the matrix-flattening method (or the original matrix). This difference between the two methods increases as the difference in the distribution of each vector increases. (c) and (d) show an example (with random values) where the distribution of each vector is clearly different.

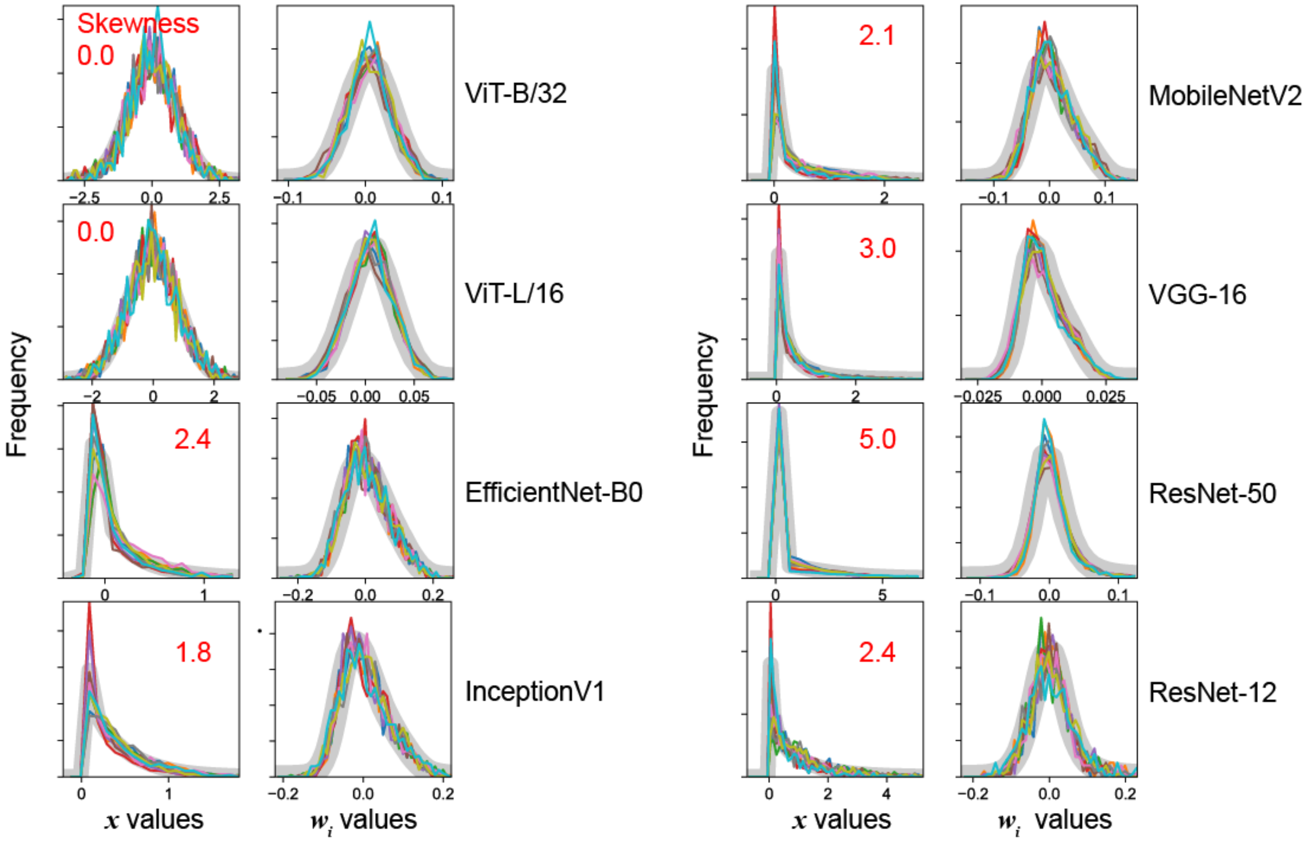

Figure S2. Difference in the shape of frequency distributions of $\boldsymbol{x}$ and $\boldsymbol{w}_i$ vectors. In all CNNs (*i.e.*, except ViT), frequency distributions of $\boldsymbol{x}$ and $\boldsymbol{w}_i$ vectors are right-tailed and bell-shaped, respectively. The $\boldsymbol{x}$ distribution of ResNet-50 shows the most prominent right-tail shape, which is considered to be one of the major causes of the large interference observed in Figure 4 (the red numbers displayed in each plot for $\boldsymbol{x}$ values indicate the skewness).

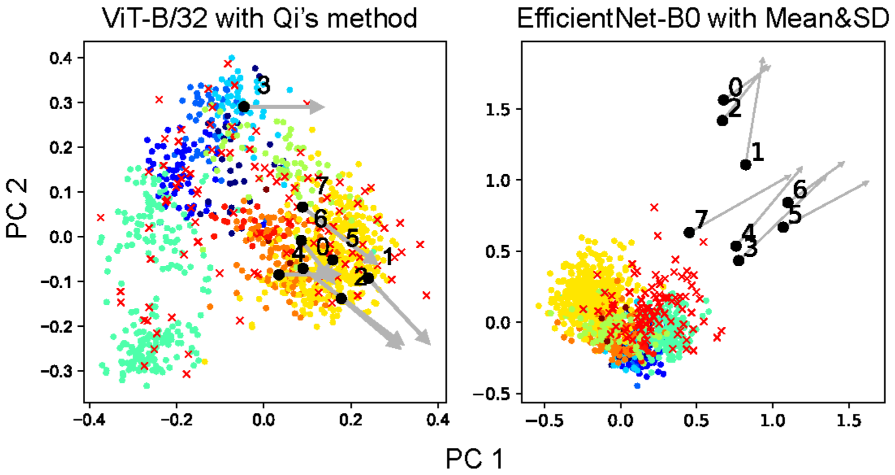

Figure S3. Principal component analysis of weight vectors (Figure 5 in the main text) in the case of ViT-B/32 with Qi's method (left) and EfficientNet-B0 with DONE(III) (right).

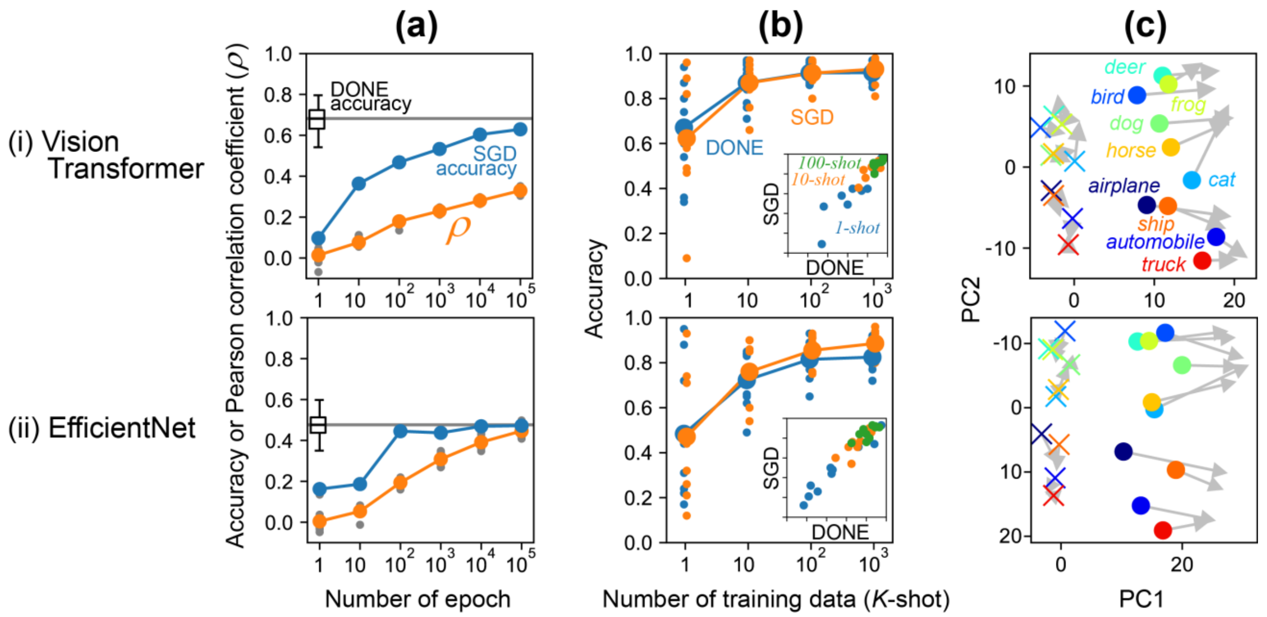

Figure S4. Transfer learning tasks of DONE. Transfer learning is not a recommended task for DONE in practical uses, but it is convenient for the understanding. Accuracy of DONE should be the lowest and baseline in one-shot learning method, which is expected to be comparable to that by using basic optimization methods. To demonstrate it, we compared the accuracy between DONE and stochastic gradient descent (SGD), one of the simplest optimizers, in multi-class classification of CIFAR-10. Specifically, we eliminated the final dense layer of DNNs, froze the other parameters, and created the weight matrix $\boldsymbol{W}$ for the 10-way classification of CIFAR-10.

In one-shot learning by DONE, one image was input from each of the 10 classes, and then each $\boldsymbol{x}$ was converted to each $\boldsymbol{w}_j$ vector to construct a new $\boldsymbol{W}$ matrix, which contains ten $\boldsymbol{w}_j$ vectors. We constructed 100 models by using 100 sets of 10 images (1 image/class). We evaluated the accuracy by using all 10,000 test images of CIFAR-10 (in (a), boxplot at epoch 1). The median accuracy of the 100 models was 68.2% and 47.6% in ViT and EfficientNet, respectively (gray line).

In one-shot learning by SGD, we used the set of 10 images that provided median accuracy in DONE, as a standard-images set. As a standard setting, the cross entropy error was minimized using the learning rate 0.01 (Tensorflow default) by increasing the number of epochs (in (a), blue circles). We found the obtained accuracy by enough epochs was similar to that obtained by DONE with both backbone DNNs. We also found that $\boldsymbol{w}_j$ vectors became similar in terms of Pearson correlation coefficient between DONE and SGD optimization as the epoch is increased (gray circles for each class, orange circles for the mean). Note that we here used a basic setting for the optimization just for comparison, but of course learning efficiency can increase by tuning hyperparameters such as increasing the learning rate. In actual use, it may work well if the initial value of $\boldsymbol{W}$ is created by DONE from one or several training data before the optimization.

We also tested the case of 10, 100, and 1000-shot learning, adjusting the number of epochs in SGD so that the number of inferences was 10,000 (i.e., 10 epochs for 1000-shot learning) with learning rate 0.1. The results are shown in (b). The small circle is the accuracy for each class, and the large circle is the average value. The inset graph is the relationship between the accuracy of each class obtained by DONE and SGD. Both methods show similar increase in accuracy (circle with line) and a difficult class for DONE was also difficult for SGD optimization (inset). SGD optimization outperformed DONE when the number of training data was large. This is probably because SGD optimization can make fine adjustments between multiple $\boldsymbol{w}_j$ vectors while each $\boldsymbol{w}_j$ vector in DONE is determined independently.

Also, PCA (in (c)) shows $\boldsymbol{w}_j$ of DONE (circles) and SGD (crosses) were somehow different in PC1 and similar in PC2, in both backbone DNNs. Increasing the number of training data made it move further apart on PC1 (gray arrows: the initial and terminal points indicate the results of one-shot and 1000-shot learning, respectively). Thus, some similarity and differences between DONE and SGD optimization were observed in the transfer learning task.

As above, immediate use of DONE (i.e., without optimization) has a similar effect to running a simple optimization (although DONE is recommended not for transfer learning tasks but for class addition tasks). Similar results are also shown in the previous paper (Qi, Brown, and Lowe 2018), because there is not much difference between DONE and Qi's method other than the interference at class-addition tasks.
